# Serum sIgG4, but not sIgA, is involved in induced and natural tolerance to egg allergens

**DOI:** 10.1186/2045-7022-3-S3-O6

**Published:** 2013-07-25

**Authors:** M Vazquez-Ortiz, M Pascal, L Alsina, M Piquer, J Lozano, MA Martin-Mateos, AM Plaza

**Affiliations:** 1 Paediatric Allergy and Clinical Immunology Section, Hospital Sant Joan de Deu, Barcelona, Spain; 2 Immunology Department, Hospital Clinic, Barcelona, Spain

## Background

Immune mechanisms underlying oral immunotherapy (OIT) for food allergy are poorly understood and might be mimicking those related to the development of natural tolerance. The role of sIgG4 and sIgA in food allergy and tolerance is controversial [1-3]. We aimed to study the specific humoral immune response involved in natural and induced tolerance to egg proteins.

## Methods

Patients. We recruited children aged 5 to 16 years with previous history of IgE-mediated egg allergy, skin prick test ≥3mm and sIgE >0.35 to ovalbumin (OVA) or ovomucoid (OVM). Egg allergy or tolerance at inclusion was assessed through a double-blind placebo-controlled egg challenge. Allergic children were divided in 2 groups: G1 underwent OIT with raw egg white, whereas G2 continued avoiding egg. Tolerant children constituted group G3. Additionally, 2 groups of atopic (nut-allergic, G4) and non atopic children (G5) were included. Serum sIgE, sIgG4 and sIgA to OVA and OVM were determined at baseline (T0) in all groups, and repeated 9 months after in G1 and G2. A second DBPCFC was performed in G2 in T1. Comparisons among groups were performed through non parametrical tests using SPSS 19.0. 2-tailed p<0.05 was considered significant.

## Results

Number of children recruited: G1 (28); G2 (24); G3 (21); G4 (10); G5 (10). sIgE decreased and sIgG4 increased in desensitized children (G1) 9 months after OIT-start, but not in egg-allergic controls (G2) (p>0.001). 4 children in G2 developed spontaneous tolerance over follow-up. In them, sIgE decreased and sIgG4 increased in T1 versus T0. Similarly, sIgE was lower and OVA-sIgG4 was higher in transient egg-allergic (G3) than in persistent egg- allergic individuals (G1 plus G2 in T0) (p<0.05). Atopic egg-tolerant children (G4) had higher sIgG4 than persistent egg-allergic, transient egg-allergic and non atopic children (p<0.05). No changes in sIgA were detected in desensitized children or in those 4 developing natural tolerance over follow-up. No differences were found in sIgA among the 5 groups.

## Conclusion

The humoral immune mechanisms involved in natural and induced tolerance to egg allergens seem to be similar. Serum sIgG4, but not sIgA, is involved in induced tolerance in egg-allergic children, as well as with natural tolerance in transient egg-allergic and atopic individuals.

## Disclosure of interest

None declared.
